# Online Health Information Seeking: An Italian Case Study for Analyzing Citizens’ Behavior and Perception

**DOI:** 10.3390/ijerph20021076

**Published:** 2023-01-07

**Authors:** Alessia D’Andrea, Patrizia Grifoni, Fernando Ferri

**Affiliations:** Institute for Research on Population and Social Policies, National Research Council, 00185 Rome, Italy

**Keywords:** health information, COVID-19, information-seeking behavior, fake information

## Abstract

This study aims to understand people’s behavior when searching for online health information (and COVID-19 information) and their perception of the trustworthiness and credibility of the searched information, the actors, and sources used to obtain it. A questionnaire addressed to people who permanently live in Italy between ages 19 and 60 has been used to collect data. Data extracted from the analysis are reassuring from the point of view of trust and credibility both in the actors and in the sources used to obtain information on health and COVID-19. A correlation between the analyzed individual features, the online health information-seeking behavior, and perception resulted from the analysis. The study also underlined a positive correlation between the perception of the influence of information on the knowledge of health problems and the ability to identify false online health information, and between the experience in detecting false health online information and the ability to detect it. Finally, a positive correlation also resulted between the experience in finding online health information and the experience in finding false COVID-19 information.

## 1. Introduction

In the past, people obtained health information mainly by consulting a healthcare professional, reading books and handbooks, or exchanging experiences with their friends. Information and communication technologies (ICTs) changed these behaviors, increasing the use of the Internet for accessing health information and services (therapies, health products, medical organizations, etc.) [1,2]. The Internet potentially facilitates information searches on health and health services; it may support patients’ choices and guarantees convenience and anonymity. However, the rapid diffusion of online health information seeking also implies risks, including finding fallacious and misleading information [3]. The spread of false, erroneous, and/or misleading information in non-official communication represents a strongly disruptive and dangerous phenomenon for health, particularly during a health emergency such as the COVID-19 pandemic. The proliferation of these kinds of information can harm people in several ways, leading them to wrong self-diagnosis or damaging treatment attempts. Moreover, they can make patients less willing to follow the doctor’s and, in general, expert’s advice. There is also the risk of financial damages if a patient decides to buy over-the-counter medication or equipment based on bad advice from websites [4]. The individual capacity of assessing online health information and sources identifying fake information, in general, plays a very important role in this scenario. The assessment is influenced by the user’s perception of the information’s trustworthiness and credibility.

Different studies have been carried out to analyze the individual features affecting online health information-seeking behavior and perception (in terms of the judgment of trustworthiness and credibility they give to online health information). However, in the existing studies, only some of these individual features are analyzed. In fact, as shown in Appendix A and Appendix B, some of the existing studies analyzed the individual features affecting online health information-seeking behavior, while other studies analyzed the individual features affecting online health information perception.

The paper tries to give contributions to the existing literature by providing an integrated analysis of the individual features affecting both online health information-seeking behavior and perception. Moreover, the paper analyzes the experience/ability in detecting false information.

The research questions guiding this study are the following: 

RQ1: What are the individual features characterizing the online health information seeking behavior?

RQ2: What are the individual features characterizing the perception of online health information seekers?

RQ3: Is there a correlation between the perception of the influence of information on health problem knowledge and the ability to identify false information?

RQ4: Is there a correlation between experience in detecting false online health information and the ability to detect it?

RQ5: Is there a correlation between experiences in finding false online health information and experience in finding false online COVID-19 information?

For the analysis, a questionnaire has been administrated using the EUSurvey platform (https://ec.europa.eu/eusurvey/home/welcome/runner, access on access on 5 June 2021), an online survey management tool to create, publish, and manage questionnaires and other interactive forms in most web browsers.

In our analysis, we were interested in the correlation between some answers to provide a picture of the situation, even if it did not have statistical significance due to the number of answers received. We decided to use Pearson’s coefficient, as Pearson’s correlation coefficient is found to be appropriate for measurements taken from an interval scale, according to Choi et al. [5]. In this respect, see also the response from Abdulvahed Khaledi Darvishan in the ResearchGate post (https://www.researchgate.net/post/Which-correlation-coefficient-is-better-touse-Spearman-or-Pearson, access on 20 December 2022).

The questionnaire has been filled out by people who live permanently in Italy and are aged between 19 and 60. The paper is structured as follows. Section 2 gives an overview of studies on online health information seeking and perception. Section 3 describes the structure of the questionnaire, and in Section 4, the results are described in detail. In Section 5, a discussion of the results is provided. Finally, Section 6 concludes the paper.

## 2. Background

This section provides a background from the literature review. We searched scientific papers on the Google Scholar database that analyze people’s behavior in seeking and using online health information. The search queries contained the following categories and keywords: behaviour (find, search, seek, access, retrieve, perception), place (Internet, online, web), object (information, fake), and attribute (health). The initial search was performed in January 2021. We searched for English-language open-access articles, with publication dates from 2016 to 2021. We only reviewed articles published in the last 6 years to make sure the findings of the literature are up-to-date. We adopted a series of inclusion and exclusion criteria. The inclusion criteria were (i) papers on health-related contexts, (ii) papers describing people’s behaviors (e.g., seeking and using online health information), (iii) papers describing people’s perception of online health information, (iv) papers published in a peer-reviewed journal or conference proceedings, and (v) papers written in English. Our exclusion criteria were (i) papers that did not pertain to a health-related context and (ii) papers not written as full papers (e.g., abstracts, posters, or letters).

Within the 100 papers retrieved, 18 papers were selected according to our inclusion and exclusion criteria (see Appendix A and Appendix B). In the following sub-section, a short description of these papers classified according to online health information seeking and perception is provided.

### 2.1. Online Health Information-Seeking Behaviour

The studies belonging to this class aimed to analyze the individual features affecting online health information-seeking behavior. Interest in the Internet as a search tool for health-related information represents a global trend [6] The profile of online health consumers includes patients, their families/friends, and people who purposely seek health-related information online to pursue good health or lifestyle [7]. There has been a continuous increase in online health information-seeking activities lately [8]. A recent survey conducted in 2020 (https://ec.europa.eu/eurostat/web/products-eurostat-news/-/edn-20210406-1, accessed on 28 April 2022) shows that generally, 55% of Europeans (16–74 years old) have sought health-related information online, with a 21% increase since 2010. In particular, the percentage of online health information seeking has reached over 70% in Finland, the Netherlands, Germany, and Denmark. The survey also shows that a similar situation is observed in the US context.

Health information seeking is associated with a wide variety of many individual factors. Attention is given in particular to the gender dimension, which is frequently reported as a relevant factor characterizing the analysis of online health information-seeking behavior. The studies provided in [9,10] underlined differences between women and men in the use of online resources for health information purposes, while no difference between men and women emerged from the study provided in [11]. In particular, results from [9,10] point out that females use the Internet to seek health information more than males. In particular, according to [9], females are more inclined to engage more frequently in searching for health information mainly for informational support, while men tend to be driven by purely informational goals. In addition to the gender dimension, other two significant factors (age and technology literacy) affecting online health information-seeking behavior emerged from the study provided in [10]. According to the authors, people over 46 years old seek significantly less online health information than other age groups; this is also because, usually, they have a lower digital technology literacy level and less experience. Similar results resulted from other studies, such as [12], where elderly people were found to have more difficulties than other groups of people per age in accepting and using technology. An interesting study is provided in [13], in which the behavior in seeking information on and help with drug-related issues among young users in Slovenia is analyzed to contribute to developing guidelines and critical recommendations for effective online interventions.

Recent studies carried out in [14,15,16] offer a more complete description of the health information seekers’ profiles. The studies underlined that online health information seekers are more likely to be women, younger, and have a higher educational level. In addition to these attributes, in [14,15], higher household income is added, while in [16] the frequent use of the Internet is noted. Differently, [9,10] and the study provided in [11] revealed that online health information seeking among men and women was generally similar, with exception of health status. The study is positively associated with online health-seeking (for women only), the reporting of poor health, and the presence of two chronic diseases. Similar results, even if not related to women only, emerged in [17]. The analysis underlined significant predictors of online health information seeking to have a chronic medical condition associated with the use of the Internet several times a day. Another example in this sense is the study provided in [18], in which lower levels of self-rated health and higher levels of psychological distress are significantly associated with higher odds of online health information-seeking behavior. The study supports the idea that individuals’ low levels of self-rated health and high levels of perceived distress make people search for online health-related information to cope with health-related concerns and distress.

In addition to gender, age, digital technology literacy, and health status are among the factors affecting online health information seeking, and there is also the educational level. According to [7,19], as the level of education increases, the tendency to seek health information on the Internet also increases.

### 2.2. Online Health Information Perception

The studies belonging to this class aimed to analyze the individual features affecting online health information perception (in terms of the judgment of trustworthiness and credibility they give to online health information). As the quality of online health information remains questionable, there is a pressing need to understand how people perceive this information. Several studies underlined individual-related factors that influence the judgment of the trustworthiness and credibility of online health information. The study provided in [20] focused on the nature of Internet users in terms of prevalence and their characteristics, such as gender, age, and socioeconomic status, analyzing the association between the use of the Internet for health care information and key outcomes, including patient confidence in providers and perceived access to health care. The authors showed that the factors affecting the relationship between online health information seeking and perceived health care quality varied significantly across age, education, gender health status, and level of satisfaction concerning their primary care use.

Another important factor influencing the perception of online health information is ethnicity. A study that analyzed the relationship between confidence and trust in health information sources with ethnicity is provided in [21], where black respondents, relative to white, claimed to have high confidence in their ability to attain health information. Furthermore, some researchers gave attention to health literacy as another factor affecting trust in online health information. According to [22], people with limited health literacy had higher rates of using and trusting sources, such as social media and blogs, which might contain lower-quality health information compared to information from healthcare professionals. These results have been also confirmed in the study provided in [23], where low health literacy has been associated with an enhancement of people’s vulnerability. Moreover, according to Zhang et al. [24], people with higher eHealth literacy gain benefits from health information, including improved self-management of healthcare needs and more effective interactions with their doctor. An interesting result is also provided in [25], where eHealth literacy is associated with a greater perceived trust in online health communication information sources; the relationship significantly varies by gender and age.

## 3. Materials and Methods

The extracted factors resulting from the literature review guided the definition of the different sections of the questionnaire targeted at people living in Italy aged between 19 and 60. The individual features shown in Table 1 have been considered for the analysis.

The working position was considered instead of the economic status, while race/ethnicity was excluded, as the study focused on Italian citizens. We measured digital technology literacy by considering the frequency of using the Internet for searching online health information.

Participants were invited to provide information related to their behavior in searching online health information, specifically on COVID-19, and their perception of trustworthiness and credibility toward searched information and the actors and sources used to obtain it. The survey started in June 2021 and ended in January 2022. Participation in the study was voluntary. Participants were provided with a link that directly connected them to an explanation of the aim of the research, the informed consent, and the online survey (https://ec.europa.eu/eusurvey/runner/Survey_2021, access on 5 June 2021). The survey did not include any information enabling the respondents’ identification, did not intercept the IP number, and did not memorize cookies. It was compliant with all laws at the national and European levels, and the general regulation for the protection of personal data; No. 2016/679 (GDPR). The questionnaire consisted of thirty-two items grouped under six different section headings labeled from A to F. Section A of the questionnaire comprised elicited personal information of respondents, including age, gender, civil status, level of study, region of residence, and employment. Questions in Section B aimed to collect information on the participant’s perception of their health state and the preferred actors (i.e., doctors, relatives, friends, and pharmacists) to contact for discussing and receiving information and clarifications related to their health problems. Participants were also asked to indicate their trust in information obtained from these actors. Section C contains questions to collect information on access and use of the Internet for searching for health information and the frequency of findings. Moreover, respondents are asked about the kind of searched information, the sources used for searching, as well as the motivations for their search. In section D, the perception of online health information, the acquired knowledge, and the perceived risks are analyzed. Moreover, participants were asked about their experience in finding online fake information and their ability to distinguish it. In Section E, the use of online health information and the consequent actions taken are analyzed. Finally, in Section F, questions sought information on access and use of the Internet for searching for COVID-19 information, and the frequency of findings is analyzed. The kind of searched information, the sources used for searching, as well as their experience with fake information, are also analyzed.

## 4. Results

**Figure 1:**
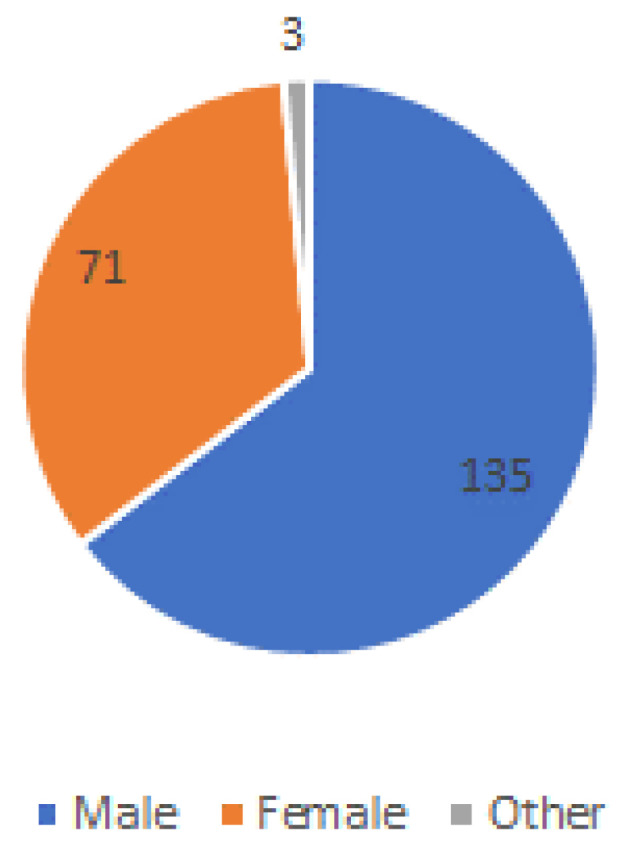
Respondents by gender.

The total number of respondents to the questionnaire was 209. A total of 135 (64.6%) were female, 71 (34%) were male, and 3 (1.4%) respondents selected the option “other” (see Figure 1).

**Figure 2:**
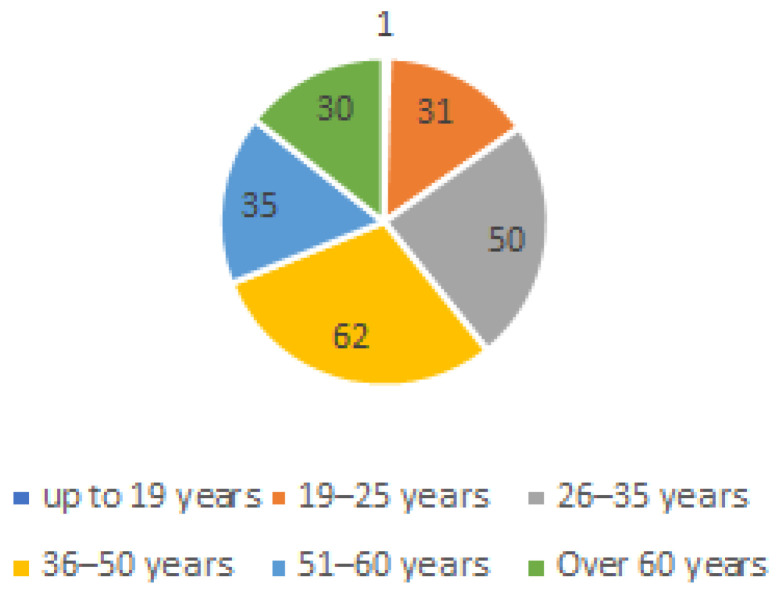
Respondents by age.

The most populated group of respondents, 62 (29.7%), were aged from 36 to 51, followed by 50 (23.9%) aged from 26 to 35. A total of 35 of them (16.7%) belong to the category between 51 and 60 years, followed by 31 (14.8%) aged between 19 and 25 years and, finally, 30 (14.40%) were over 60. Respondents who were aged 19 years were only 0.5% (1) of the total respondents (see Figure 2).

**Figure 3:**
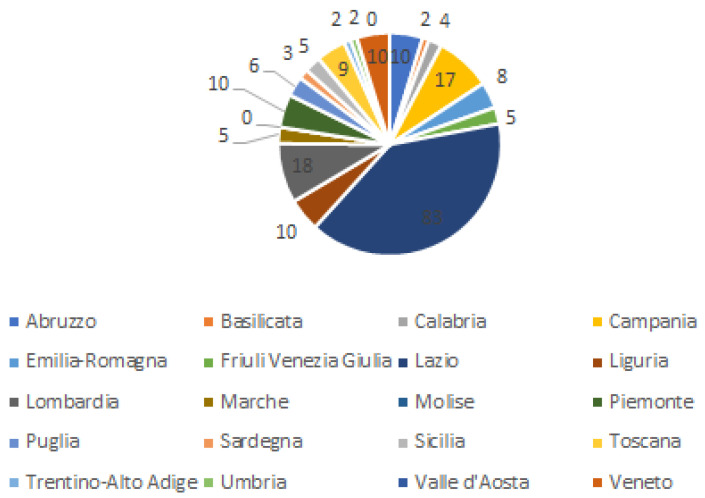
Respondents by region.

In addition to information on the basic demographic variables of gender and age, data regarding the regions where they live, educational level, and working position of respondents have been collected. The majority of respondents, 83, are living in the Lazio region, followed by respondents from Lombardia (18), Campania (17), Abruzzo, Piemonte, Liguria, and Veneto with an equal number of respondents (10), followed by Toscana (9), and Emilia-Romagna (8). Only a few respondents indicated they live in the other Italian regions of Basilicata, Trentino-Alto Adige, and Umbria (2), Calabria (4), Friuli Venezia Giulia, Marche, and Sicilia (5), Puglia (6), and Sardegna (3). No respondents stated to live in the Molise and Valle d’Aosta regions (see Figure 3).

**Figure 4:**
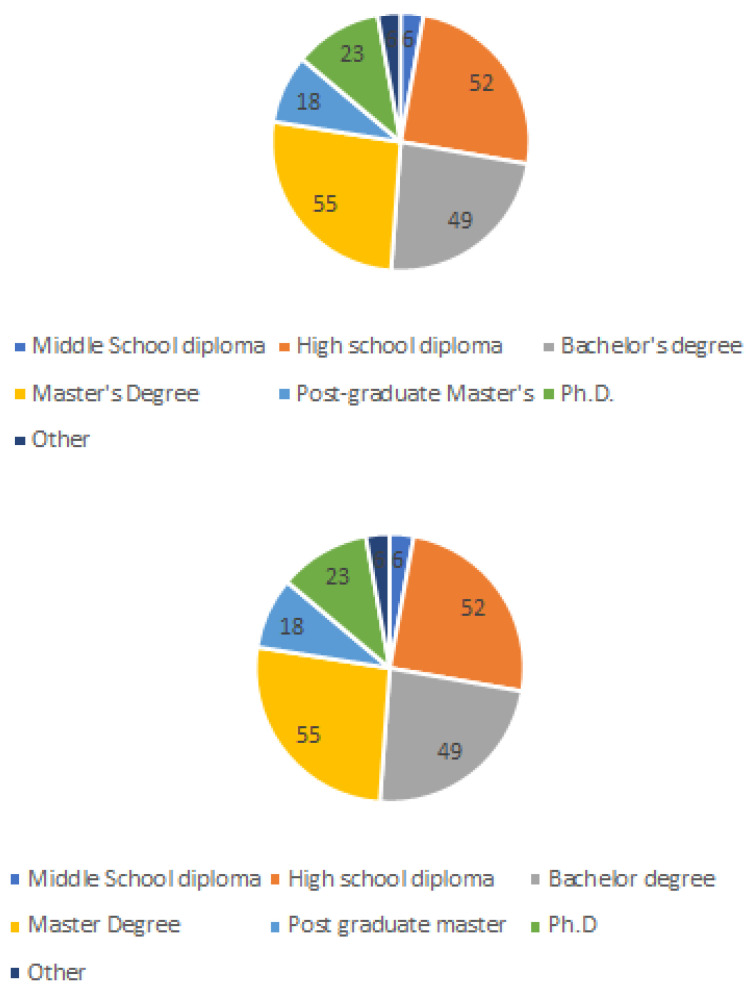
Respondents by education.

Concerning the education level, the most populated group of respondents had a 5-year bachelor’s degree (55), followed by respondents with a high school diploma (52), and bachelor’s degree (49). A total of 23 respondents had a Ph.D., while 18 had a Master’s degree. Only six respondents had a grade 2 or another education level (see Figure 4).

**Figure 5:**
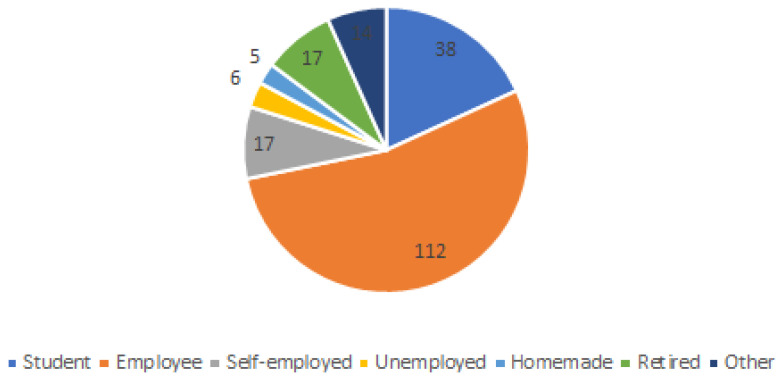
Respondents by working position.

Finally, considering the job position, the majority were employed (112), followed by students (38), self-employed and retired (17), others (14), unemployed (6), and homemade (5) (see Figure 5).

### 4.1. Section B: General Information

This section of the questionnaire aimed to collect information on the participant’s perception of their health status, the sources they use to obtain information related to health problems (i.e., doctors, relatives, friends, pharmacists, and websites), and their trust in these sources.

#### 4.1.1. Health Status

**Figure 6:**
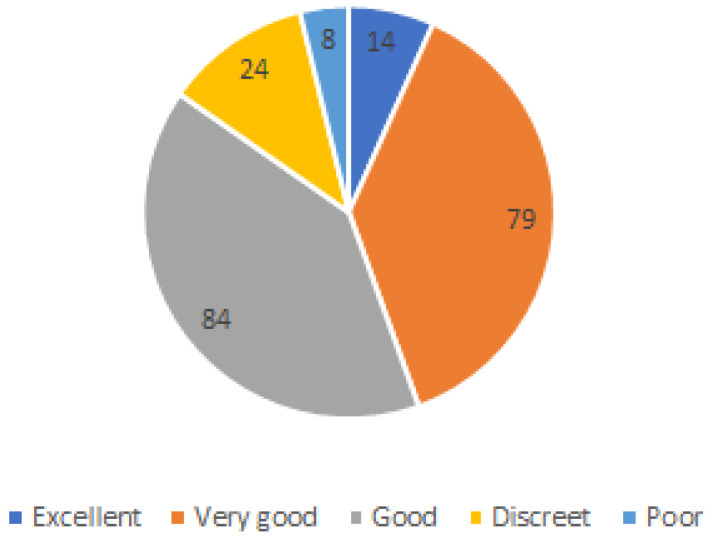
Respondents by perceived health status.

It is important to say that perceived health status is difficult to interpret because responses may be affected by the formulation of survey questions and responses, and by social factors. With this limitation in mind, the analysis shows that a majority of respondents (84) report being in good health, followed by 79 respondents with very good health status, 24 with discreet, and 14 with excellent health status, while the minority of respondents said to have a poor health status (as shown in Figure 6).

Among the respondents with good and/or very good health, the majority are female (55 and 51, respectively), the same is true for respondents with a discreet and poor health status, where the majority is represented by females (16 and 6, respectively). An equal number of answers have been obtained from females and males with excellent health status (7).

On considering the age dimension, relevant data are due to the high range age (51–60 years and over 60 years old) of the majority of respondents with good health (26), very good health (22), discreet (17), and excellent health (4), while the majority of responding with poor health (15) are between 36 and 50 years.

#### 4.1.2. Sources to Obtain Information Related to Health Problems and Perceived Trust

Four different actors/sources to obtain information related to health problems have been considered within the questionnaire (i.e., the family doctor, the pharmacist, relatives and friends, and websites). In Table 2, the answers given by respondents with percentages are shown.

The family doctor is the favorite actor to whom the respondents turn very often (14%), often (26%), and sometimes (32%) to receive information and clarifications relating to health. A good percentage of respondents (34%) said they sometimes use websites to search for health information, but at the same time, the majority of respondents (37%) said they rarely use them. The same percentage of respondents (37%) rarely turn to the pharmacist, while the actors to whom the majority of respondents never turn (30%) are relatives and friends. These data are only partially in line with those relating to confidence (see Table 3). The family doctor, who is the preferred actor for receiving health information, is also the actor that respondents have more trust. A total of 16% of respondents stated to have very much trust and 37% much trust. The pharmacist is given sufficient confidence by the majority of respondents (48%). Little trust is given to websites (46%), followed by relatives and friends, toward which respondents have little trust (38%) or no trust (13%).

### 4.2. Section C: Search for Online Health Information

This section contributes to identifying the individual features characterizing online health information seekers’ behavior (RQ1). The access and use of the Internet for searching for health information and the frequency of findings are analyzed. Moreover, an analysis of the kind of searched information, the sources used for searching, as well as the motivations are carried out.

#### 4.2.1. Access and Use of the Internet for Searching for Health Information and the Frequency of Searching

**Figure 7:**
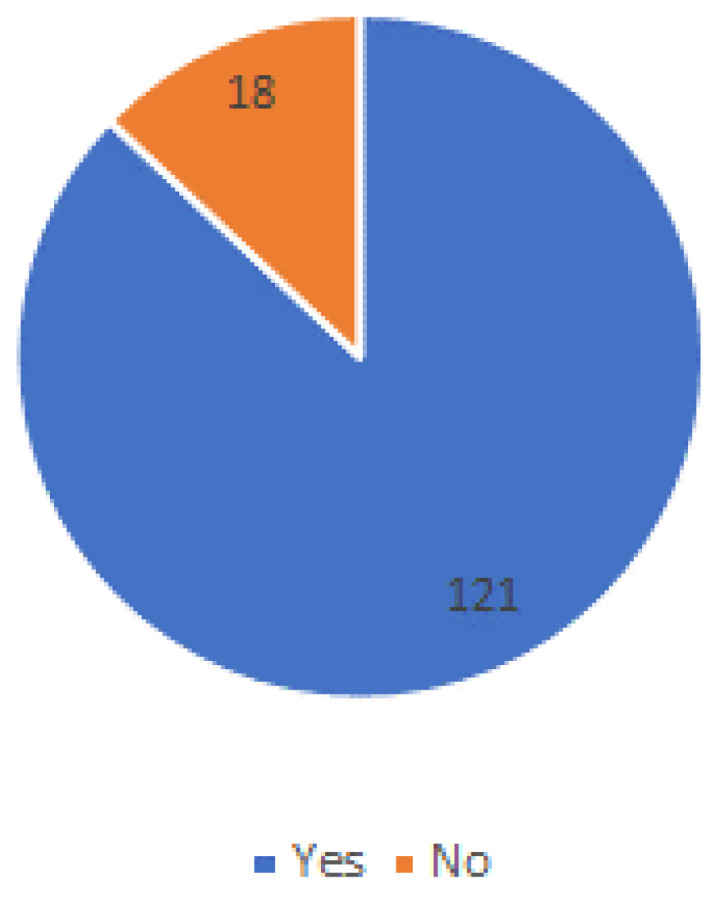
Access and use of the Internet.

The Internet is a very helpful resource the majority of respondents (191) use to find health information (see Figure 7).

However, despite this extended use, the frequency of spending time with respondents searching online health information is limited. The majority of respondents (82) seek online health information once a month, followed by 63 respondents no more than 2–3 times a year.

#### 4.2.2. The Kind of Searched Information, Websites, and Media Used for Searching for Information and Motivation

Health information is any personal information about health or disability. It includes information or opinion about illness, injury, treatment, disability, etc. A list of fourteen types of online health information has been suggested to respondents who have been asked to indicate how often they search for each of them. The list of online health information is shown in Table 4.

The booking visit and/or clinical exams is the most searched information from the majority of respondents that stated searching it very often (11%) and/or often (37%), followed by information on the specific diseases that are searched very often by 9% of respondents, often by 30%, and sometimes by 31% of respondents. A relevant percentage of respondents (32%) stated to search sometimes information on hospitals, clinics, and analysis laboratories, while 30% sometimes search for information on correct lifestyle and therapies/treatments. Concerning the information on therapies/treatments, it is also important to underline that a high percentage of respondents stated searching them rarely (32%), followed by side effects of therapies with, 35% of respondents that search it rarely. Less searched is the information on transplants for the 69% that never search it and the 19% that searched it rarely. Respondents were also asked to indicate the source from which they look for the different kinds of information. With the rapid explosion of different online information sources, one of the critical issues raised by experts involves the credibility of health web sites. This concern relates to the extent to which consumers are obtaining their information from web sites that are not qualified to provide health information. Data extracted from the analysis are reassuring from the point of view of the credibility of the sources used by respondents for searching for online health information. The major of respondents (10%) stated to use very often the website of the Ministry of Health, and 14% use it often. Additionally, the websites of other entities of the National Health System are used very often by 7% of respondents, often by 18%, and 31% sometimes. A relevant percentage of respondents (32%) also stated to use online medical journals for searching for online health information, while fewer used sources of the websites of pharmaceutical companies, which were used rarely by 34% of respondents and never used by 49% (Table 5).

Many reasons lead people for searching online health information (see Table 6). The possibility to obtain information quickly is the main reason why the respondents (20%) give very much importance, while 34% give much importance, and 30% give enough importance. Enough importance is also given by the majority of respondents (33%) to the possibility to have an opinion different from the family doctor and obtaining much more information (31%).

### 4.3. Section D: Perception of Online Health Information

This section contributes to identify the individual features characterizing online health information seekers’ behavior (RQ2). Moreover, the possible correlation between the perception of the influence of information on the knowledge of health problems and the ability to identify false online health information (RQ3), as well as the correlation between the experience in detecting false health online information and the ability to detect it (RQ4), are also analyzed. Finally, a possible correlation between the experience in finding online health information and the experience in finding false COVID-19 information (RQ5) are also evaluated.

The acquired knowledge from online health information seeking and the perceived risks are analyzed. The seeking of online health information allows improving the knowledge of health problems for the majority of respondents (96 respondents); on the other hand, a no less significant number (56 respondents) defined it as having little impact on their knowledge.

These data are supported by the majority of respondents (129), who declared that seeking online health information increases the exchange of information with their doctor.

The doctor–patient relationship can allow reducing the risks that users can occur during online health information seeking and which can be hazardous to their health, as shown in Table 7. Among the risks, the majority of respondents underlined the incorrect and/or late self-diagnosis; 38% of respondents give this risk very much and/or 33% much relevance, followed by the risk of the state of anxiety toward the interpretation of a symptom, to which 33% of the respondent that give this rick very much and/or 30% much relevance. An important data concern is the possibility of finding false information; it is important to underline that even if 33% say this risk has been considered as the least relevant, 22% of respondents give it little importance and 2% no importance.

This aspect is in line with answers given by respondents to the question on the experience of having found online fake information.

**Figure 8:**
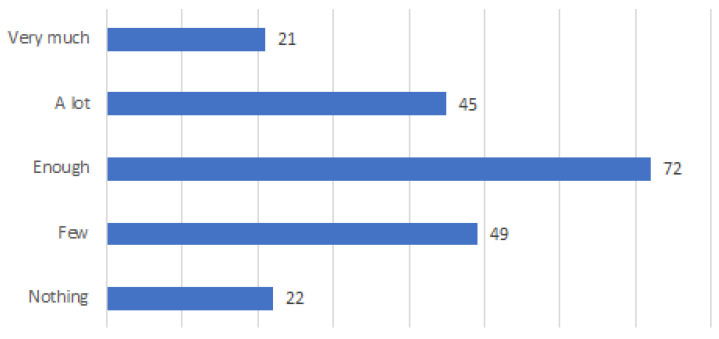
Finding false online health information.

**Figure 9:**
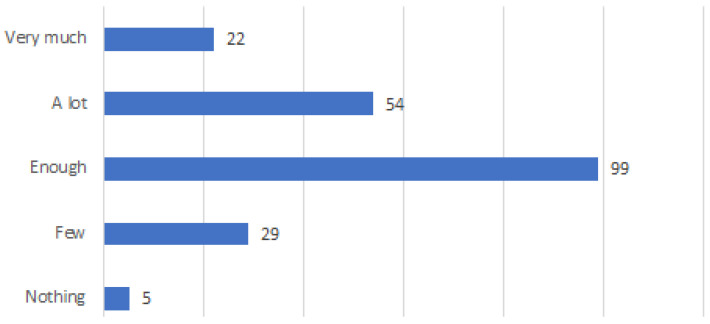
Ability to distinguish true and false online health information.

Even the majority of respondents (72 respondents) declared to have enough experience, while 22 respondents did not experience it. However, an interesting number of respondents (45) declared to have a lot of experience and 21 respondents had very much experience (see Figure 8). A little experience has been also declared by a considerable number of respondents (49), while 22 respondents have not had any experience. In terms of the ability to identify fake news, the majority of respondents (99) were confident of being enough able to do so. Only five respondents admitted to not having the confidence to differentiate fake news from non-fake news (see Figure 9).

Among elements characterizing false information factors, the most identified were the exaggerated and high-sounding titles, to which 36% of respondents give very much relevance and 31% much relevance, followed by the URL very similar to that of an existing site, to which 28% of respondents give very much relevance, 33% much, and 29% enough relevance. Enough relevance is also given to the abnormal text formatting by 30% of respondents, while less relevance is given to the typing errors by 22% of respondents that give it little reliance and 6% no relevance (see Table 8).

Respondents consider minor risks the typing errors (59 participants) and abnormal text formatting (50 participants). Respondents were also asked to express their ideas on the most relevant aspects for assessing the credibility of online health information (see Table 9). Among them, respondents consider the most relevant aspect the certified reliability of the information source (e.g., the Ministry of Health), with 57% of respondents that give it very much and 23% much relevance. Good relevance is also given to the opinions expressed by healthcare professionals with 26% of respondents giving it very much, 35% much, and 31% enough relevance, and the explanation of the reasoning underlying the advice, with 37% of respondents that give it enough importance. Low relevance is given to the number of opinions expressed on the same issue with, 27% of respondents that give it little relevance and 11% no relevance.

Among the criteria, they are used to determine if the information is false if some of them have been suggested by respondents. In Table 10, an extraction of free answers given by respondents is shown.

Three positive correlations resulted from the study (as shown in Table 11). The first correlation is between the perception of the influence of information on the knowledge of health problems and the ability to identify false online information. The second correlation is between the experience in detecting false health information online and the ability to detect it. Finally, a positive correlation resulted between the experience of finding false health information online and the experience of finding false COVID-19 information.

### 4.4. Section E: Use of Online Health Information

In this section, respondents were asked about their behavior after searching for online health information (see Table 12). The majority of the respondents set an appointment with the family doctor and discussed the online health information with the family doctor. More in detail, 11% stated that they set very often an appointment with the family doctor, while 20% did so often and 31% sometimes. A total of 7% of respondents very often discussed online health information with the family doctor and 34% did so often. A relevant percentage of respondents (23%) stated they changed their lifestyle without discussing it with the family doctor. The least adopted behavior is a change of medication without discussing it with the family doctor, with 84% of respondents stating that they never adopted this behavior.

This represents a reassuring result that reinforces the importance use and completing online health information seeking in synergy with a trust-based doctor–patient interaction. This is also confirmed by the majority of respondents (194) that declared to have never (or rarely) canceled an appointment with the family doctor. Moreover, respondents were also asked about their actions when they receive fake information; it is important to underline that for this question, they could give more than one answer.

Ignoring the news without sharing it is the behavior that received the most answers (134), followed by comments on the news expressing doubts about its unreliability and the action to talk about the information with the family doctor to obtain an opinion about it (35 answers, respectively). Only nine answers were for the sharing of the information on social media for preventive purposes, highlighting the unreliability, and one answer was for the sharing of the information on social media to obtain an opinion from their friends.

### 4.5. Section F: Search for Online COVID-19 Information

In this section, the access and use of the Internet for searching for COVID-19 information and the frequency of findings are analyzed. The Internet is a very helpful resource for searching for COVID-19 information for the majority of respondents (195).

These data have been also confirmed by the frequency of spending time searching for COVID-19 information online. The majority of respondents (64) seek online health information several times a week, while 17 did so several times a day. Interesting data are given by 36 respondents who stated searching for COVID-19 information online only once a month. The search for online information on COVID-19 improved the knowledge of the major number of respondents (114). A total of 51 respondents stated that their knowledge has increased a lot, while 18 respondents stated very much. Only twenty-three respondents stated their knowledge improved a little or not at all (three respondents).

The most searched COVID-19 searched online by respondents is the spread of COVID-19 in their own country, with 31% of respondents stated searching for it very often, 33% often, and 28% sometimes, followed by the COVID-19 containment measures in place in their own country, with 28% of respondents that stated to search for it very often, 34%, often and the 29% sometimes. The less searched information is the COVID-19 variants, with 29% of respondents stated to searching for it rarely and 9% never (see Table 13).

As for the sources used for searching online health information, the most used source for searching online COVID-19 information by respondents is the website of the Ministry of Health, with 32% of respondents stating to use it very often, 31% often, and 23% sometimes, followed by the website of the region, with 20% of respondents stating to use for it very often, 26% often, and 28% sometimes. The less-used sources are the websites of pharmaceutical companies, with 25% of respondents stating to use them rarely and 63% never, followed by the websites of associations of patients with specific pathologies, with 22% of respondents stating to use them rarely and 58% never (see Table 14).

When considering the experience in finding fake online information about COVID-19 online, the majority of respondents (78) stated to have enough experience, followed by 40 respondents that had much and 37 with very much experience. A considerable number, 33, stated to have little experience.

Moreover, respondents were also asked about their experience in finding health information that later turned out to be false. Seven respondents had little experience, while sixty-six had no experience. Eight respondents had enough experience, while fourteen respondents had much and only eight respondents had very much experience.

## 5. Discussion

As said in the introduction, the questionnaires aimed to understand the individual features characterizing the online health information seekers’ behavior and perception.

Considering RQ1, the study has shown that women were more likely than men to search for health and COVID-19 information, even if the frequency with which women and men search for online health information is once a month. Additionally, online health information seekers were aged 36–50, mostly employed, with a master’s degree, and a good health status. Having a good health status drives respondents to seek more online health information (and specifically COVID-19 information) but it is also an important condition for the perception of trust in obtaining health information from official sources (i.e., their family doctors and the official websites). The trust in family doctors (the favorite actor indicated by respondents for receiving health information) is confirmed by respondents with a very good and good perception of their state of health. The trust in the family doctor has been also confirmed by the majority of respondents that stated to make an appointment with the family doctor after searching for health information online. Data extracted from the analysis are reassuring from the point of view of the credibility of the sources used by respondents for searching for online health information. The most used source for searching online health and/or COVID-19 information is the website of the Ministry of Health. The major users of the Ministry of Health website are respondents with very good (52 respondents) and good health status (44 respondents). Considering perception (RQ2), women aged 36–59 with good health status are the seekers that found enough false online health information and COVID-19 information, and who know how to distinguish true from false information.

The absence of pressure linked to a health emergency (having a good health status) seems to suggest to people virtuous behaviors (selecting certified sources) when searching for information. Therefore, the adoption of an adequate communication plan by the authorities in charge in the event of pandemics or emergency situations assumes particular importance. The communication should be clear, transparent, and provide all information to address the situation. A communication plan of this type can contribute to establishing a trustful relationship among citizens at the institutional level helping to overcome any inappropriate behavior due to anxiety. Moreover, the use of certified sources is also connected to the high level of education. The major users of the website of the Ministry of Health are respondents with a Master’s degree (27 respondents) and post-graduate Master’s/Ph.D. (26 respondents). This implies that the level of education is crucial for better use and selection of online sources. Therefore, organizing specific training on the use of the Internet and, in particular, on its use related to healthcare issues and emergency situation management could be strategic. Knowledge is a key factor.

Three positive correlations resulted from the study. The first correlation is between the perception of the influence of information on the knowledge of health problems and the ability to identify false online (RQ3). The second correlation is between the experience in detecting false health online information and the ability to detect it (RQ4). Finally, a positive correlation also resulted in the experience of finding online false health information and the experience of finding false COVID-19 information (RQ5).

## 6. Conclusions

This study was conducted to identify individual features characterizing online health information seekers’ behavior (RQ1) and perception (RQ2) among Italian citizens, and the importance of experience (the importance of improving skill) in finding online information for identifying false information (RQ3, RQ4, and RQ5). In particular, findings revealed that gender, education, and health status were significant factors that influence online health information-seeking behavior. Considering the perception, an important result of the study is that online health information seekers consider the family doctor a point of reference both for obtaining information and for discussing the information found online. A good awareness of the importance of using official sources to search for information online and/or COVID-19 resulted from the analysis. In fact, the respondents mainly rely on official sources (in particular, the website of the Ministry of Health) for health information research. Added to this is also the respondents’ awareness of the importance of using certified information sources (e.g., the Ministry of Health) to assess the credibility of online health information. The positive correlations resulting from the analysis of RQ3, RQ4, and RQ5 highlighted an important aspect. Experience in searching online information is important for identifying false health information online, as it can contribute to improving the skill of people (especially younger people); but a useful experience needs to be fostered by training for using the Internet in a safe way, such as to help recognize unreliable information more easily. In this perspective, educational strategies should be activated at the school level to train, guide, and motivate students to adopt healthy behaviors and become more aware of the risks associated with online health information. By educating students to be savvy consumers of online health information, schools can become trusted sources for health communication and positively influence people’s health now and in the future. As students develop healthy behaviors based on factual health information, they will experience positive short-term health outcomes that can significantly influence health outcomes. This knowledge will also affect the lives of the people closest to them. This result provides an important lesson learned for policy and decision makers (education as well as the health ministry), that should focus their actions mainly on training on the correct use of the Internet, instead of only the identification of risks without any indication about the behaviors to adopt to identify them. The study only considered the variables extracted from the analyzed studies; other variables should be considered. Moreover, the study involved a small number of Italian respondents; it was limited geographically and indeed, results are not generalizable to other countries different from the Italian context.

Future studies could consider other individual factors affecting people’s behavior and perception of online health information in various geographical countries; moreover, cross-country studies comparing similarities and differences should be conducted.

## Figures and Tables

**Table 1:** Individual features considered for the analysis.

Individual Features for Online Health Information-Seeking Behavior	Individual Features for Online Health Information Perception
Gender Age Educational level Working position Health status	Gender Age Health status

**Table 2:** Actors/sources used to obtain information related to health problems.

WHO DO YOU TURN TO FOR HEALTH INFORMATION?						
		Very Often	Often	Sometimes	Rarely	Never
**The family doctor**	Number of answers given by respondents	30	54	66	45	14
	Percentage	14%	26%	32%	22%	7%
**The** **pharmacist**	Number of answers given by respondents	4	24	47	77	57
	Percentage	2%	11%	22%	37%	27%
**Relatives and friends**	Number of answers given by respondents	3	19	55	69	63
	Percentage	1%	9%	26%	33%	30%
**Websites**	Number of answers given by respondents	4	26	71	78	30
	Percentage	2%	12%	34%	37%	14%

**Table 3:** Perceived trust in actors/sources used to obtain information related to health problems.

Do you trust the information obtained from …??						
		Very Much	Much	Enough	Few	Nothing
**The family doctor**	Number of answers given by respondents	33	78	72	24	2
	Percentage	16%	37%	34%	11%	1%
**The pharmacist**	Number of answers given by respondents	6	55	100	38	10
	Percentage	3%	26%	48%	18%	5%
**Relatives and friends**	Number of answers given by respondents	2	17	84	79	27
	Percentage	1%	8%	40%	38%	13%
**Websites**	Number of answers given by respondents	1	15	76	96	21
	Percentage	0%	7%	36%	46%	10%

**Table 4:** Kinds of searched online health information.

WHAT KIND OF HEALTH INFORMATION DO YOU SEARCH FOR ON THE WEB?						
		Very Often	Often	Sometimes	Rarely	Never
**Specific diseases**	Number of answers given by respondents	18	63	64	51	13
	Percentage	9%	30%	31%	24%	6%
**Therapies/treatments**	Number of answers given by respondents	12	43	63	66	25
	Percentage	6%	21%	30%	32%	12%
**Side effects of therapies**	Number of answers given by respondents	14	37	46	73	39
	Percentage	7%	18%	22%	35%	19%
**Side effects of drugs**	Number of answers given by respondents	12	37	57	62	41
	Percentage	6%	18%	27%	30%	20%
**Alternative medicine**	Number of answers given by respondents	8	16	37	50	98
	Percentage	4%	8%	18%	24%	47%
**Experimental treatments**	Number of answers given by respondents	5	15	33	70	86
	Percentage	2%	7%	16%	33%	41%
**Purchase of drugs**	Number of answers given by respondents	6	29	34	58	82
	Percentage	3%	14%	16%	28%	39%
**Doctors and/or specialists**	Number of answers given by respondents	12	36	57	70	34
	Percentage	6%	17%	27%	33%	16%
**Hospitals, clinics, and analysis laboratories**	Number of answers given by respondents	11	50	67	55	26
	Percentage	5%	24%	32%	26%	12%
**Booking of visits and/or clinical exams**	Number of answers given by respondents	24	78	56	34	17
	Percentage	11%	37%	27%	16%	8%
**Vaccinations and cancer screening**	Number of answers given by respondents	13	42	59	63	32
	Percentage	6%	20%	28%	30%	
**Correct lifestyle**	Number of answers given by respondents	19	53	62	57	
	Percentage	9%	25%	30%	27%	
**Transplants**	Number of answers given by respondents	5	8	12	39	
	Percentage	2%	4%	6%	19%	
**Healthcare data and statistics**	Number of answers given by respondents	10	33	46	63	
	Percentage	5%	16%	22%	30%	

**Table 5:** Websites and media are used for searching online health information.

WHERE DO YOU SEARCH FOR HEALTH INFORMATION ONLINE?						
		Very Often	Often	Sometimes	Rarely	Never
**Website of the Ministry of Health**	Number of answers given by respondents	21	30	59	70	29
	Percentage	10%	14%	28%	33%	14%
**Website of the region**	Number of answers given by respondents	7	36	51	71	44
	Percentage	3%	17%	24%	34%	21%
**Website of the local health authority**	Number of answers given by respondents	9	23	57	64	56
	Percentage	4%	11%	27%	31%	27%
**Websites of other entities of the National Health System**	Number of answers given by respondents	15	38	65	59	32
	Percentage	7%	18%	31%	28%	15%
**Websites of associations of patients with specific pathologies**	Number of answers given by respondents	8	16	40	69	76
	Percentage	4%	8%	19%	33%	36%
**Websites of pharmaceutical companies**	Number of answers given by respondents	1	7	27	72	102
	Percentage	0%	3%	13%	34%	49%
**Wikipedia**	Number of answers given by respondents	7	21	52	72	57
	Percentage	3%	10%	25%	34%	27%
**Online forums on specific diseases**	Number of answers given by respondents	5	23	52	66	63
	Percentage	2%	11%	25%	32%	30%
**Online medical journals**	Number of answers given by respondents	15	23	69	53	49
	Percentage	7%	11%	33%	25%	23%

**Table 6:** Motivation for searching online health information.

WHAT ARE THE MOTIVATIONS THAT MAKE YOU LOOK FOR HEALTH INFORMATION ONLINE?						
		Very Much	Much	Enough	Few	Nothing
**Lack of time to go to the family doctor**	Number of answers given by respondents	14	20	63	56	56
	Percentage	7%	10%	30%	27%	27%
**Lack of trust in the family doctor**	Number of answers given by respondents	9	10	41	55	94
	Percentage	4%	5%	20%	26%	45%
**To obtain information quickly**	Number of answers given by respondents	42	71	63	22	11
	Percentage	20%	34%	30%	11%	5%
**Embarrassment to ask certain questions to the family doctor**	Number of answers given by respondents	4	7	29	45	124
	Percentage	2%	3%	14%	22%	59%
**To obtain much more information**	Number of answers given by respondents	20	28	65	49	47
	Percentage	10%	13%	31%	23%	22%
**To check the therapies recommended by the family doctor**	Number of answers given by respondents	12	14	36	54	93
	Percentage	6%	7%	17%	26%	44%
**To have an opinion different from the family doctor**	Number of answers given by respondents	17	29	68	47	48
	Percentage	8%	14%	33%	22%	23%

**Table 7:** Risks of searching for online health information.

WHAT ARE THE RISKS OF SEARCHING FOR ONLINE HEALTH INFORMATION?						
		Very Much	Much	Enough	Few	Nothing
**Misunderstanding of information**	Number of answers given by respondents	40	74	55	34	6
	Percentage	19%	35%	26%	16%	3%
**Incorrect and/or late self-diagnosis**	Number of answers given by respondents	79	68	40	17	5
	Percentage	38%	33%	19%	8%	2%
**State of anxiety toward the interpretation of a symptom**	Number of answers given by respondents	70	62	45	21	11
	Percentage	33%	30%	22%	10%	5%
**Suggestions of treatments/medicines not suitable for the specific clinical pathology**	Number of answers given by respondents	50	80	48	25	6
	Percentage	24%	38%	23%	12%	3%
**Possibility of finding fake information**	Number of answers given by respondents	39	51	69	45	5
	Percentage	19%	24%	33%	22%	2%

**Table 8:** Elements characterizing false information.

WHAT ARE THE ELEMENTS THAT CHARACTERIZE FALSE INFORMATION?						
		Very Much	Much	Enough	Few	Nothing
**Exaggerated and high-sounding titles**	Number of answers given by respondents	76	64	56	13	0
	Percentage	36%	31%	27%	6%	0%
**URL very similar to that of an existing site**	Number of answers given by respondents	58	68	61	16	6
	Percentage	28%	33%	29%	8%	3%
**Retouched photos or videos**	Number of answers given by respondents	60	67	52	21	9
	Percentage	29%	32%	25%	10%	4%
**Typing errors**	Number of answers given by respondents	50	45	55	46	13
	Percentage	24%	22%	26%	22%	6%
**Abnormal text formatting**	Number of answers given by respondents	46	50	63	36	14
	Percentage	22%	24%	30%	17%	7%

**Table 9:** Aspects to assess the reliability of the online health information.

WHEN ASSESSING THE RELIABILITY OF ONLINE HEALTH INFORMATION, WHICH ASPECTS DO YOU CONSIDER RELEVANT?						
		Very Much	Much	Enough	Few	Nothing
**Certified reliability of the information source (e.g., the Ministry of Health)**	Number of answers given by respondents	119	49	31	10	0
	Percentage	57%	23%	15%	5%	0%
**Ease of understanding of the contents**	Number of answers given by respondents	17	62	71	50	9
	Percentage	8%	30%	34%	24%	4%
**Explanation of the reasoning underlying the advice**	Number of answers given by respondents	28	70	78	25	8
	Percentage	13%	33%	37%	12%	4%
**Number of opinions expressed on the same issue**	Number of answers given by respondents	12	46	72	57	22
	Percentage	6%	22%	34%	27%	11%
**Opinions expressed by healthcare professionals**	Number of answers given by respondents	54	73	65	15	2
	Percentage	26%	35%	31%	7%	1%

**Table 10:** Criteria suggested by respondents to determine if the information is false.

The profound discrepancy with other sources
Outdated information
Information coming from crap sites
Information is shared by a small number of people
Lack of scientific references
Suspicious sponsorship
Use of an emphatic tone
Coming from unreliable, unverifiable, and private sources
Insufficient and illogical arguments
Vague information
Typos and misspelled organ names
A continuing contradiction regarding the subject dealt with
Denied by competent people
They were at odds with other sources
Lack of tangible arguments
In contrast with previously acquired information and personal knowledge
Published on a fake site
Not in line with recognized medical standards
In contrast with expert opinions
Too distant from other given information
Not supported by feedback from doctors
Retouched photos and grammatical errors
Exaggeration in diagnosis

**Table 11:** Positive correlations resulted from the study.

Correlation	Ability to Distinguish True and False Online Health/COVID-19 Information
Health information improves the knowledge of the health problems	Pearson correlation = 0.823 Sig = 0.087
Experience in having found false health information online	Pearson correlation = 0.904 Sig = 0.017
Experience in having found false health information online in general	Pearson correlation = 0.843 Sig = 0.037

**Table 12:** Behavior after searching for online health information.

GENERALLY, AFTER SEARCHING FOR ONLINE HEALTH INFORMATION, WHAT IS YOUR BEHAVIOR?						
		Very Often	Often	Sometimes	Rarely	Never
**Set an appointment with the family doctor**	Number of answers given by respondents	22	41	64	48	34
	Percentage	11%	20%	31%	23%	16%
**Deleted an appointment with the family doctor**	Number of answers given by respondents	1	4	10	52	142
	Percentage	0%	2%	5%	25%	68%
**Discussed the online health information with the family doctor**	Number of answers given by respondents	14	71	32	48	44
	Percentage	7%	34%	15%	23%	21%
**Changed the medication without discussing it with the family doctor**	Number of answers given by respondents	3	5	9	17	175
	Percentage	1%	2%	4%	8%	84%
**Changed the lifestyle without discussing it with the family doctor.**	Number of answers given by respondents	5	18	49	44	93
	Percentage	2%	9%	23%	21%	44%

**Table 13:** Kind of information searched online regarding COVID-19.

WHAT KIND OF COVID-19 INFORMATION DO YOU SEARCH FOR ON THE WEB?						
		Very Often	Often	Sometimes	Rarely	Never
**Spread of COVID-19 in their own country**	Number of answers given by respondents	64	68	58	13	6
	Percentage	31%	33%	28%	6%	3%
**Information on the behavior to follow**	Number of answers given by respondents	53	53	64	27	12
	Percentage	25%	25%	31%	13%	6%
**COVID-19 containment measures in place in their own country**	Number of answers given by respondents	58	71	61	12	7
	Percentage	28%	34%	29%	6%	3%
**COVID-19 variants**	Number of answers given by respondents	32	32	65	61	19
	Percentage	15%	15%	31%	29%	9%
**COVID-19 vaccinations**	Number of answers given by respondents	49	60	59	29	12
	Percentage	23%	29%	28%	14%	6%

**Table 14:** Website and media used for searching online COVID-19 information.

WEBSITES AND MEDIA USED FOR SEARCHING ONLINE HEALTH INFORMATION						
		Very Often	Often	Sometimes	Rarely	Never
**Website of the Ministry of Health**	Number of answers given by respondents	67	64	48	22	7
	Percentage	32%	31%	23%	11%	3%
**Website of the region**	Number of answers given by respondents	44	55	58	27	26
	Percentage	20%	26%	28%	13%	13%
**Website of the local health authority**	Number of answers given by respondents	19	37	36	56	60
	Percentage	9%	18%	17%	27%	29%
**Websites of other entities of the National Health System**	Number of answers given by respondents	32	46	57	46	27
	Percentage	15%	22%	27%	22%	13%
**Websites of associations of patients with specific pathologies**	Number of answers given by respondents	6	11	24	46	121
	Percentage	3%	5%	12%	22%	58%
**Websites of pharmaceutical companies**	Number of answers given by respondents	3	5	16	52	132
	Percentage	1%	2%	8%	25%	63%
**Wikipedia**	Number of answers given by respondents	4	11	29	42	122
	Percentage	2%	5%	14%	20%	59%
**Online forums on specific diseases**	Number of answers given by respondents	6	14	34	46	108
	Percentage	3%	7%	16%	22%	52%
**Online medical journals**	Number of answers given by respondents	20	25	53	41	69
	Percentage	10%	12%	25%	20%	33%

## Data Availability

The data presented in this study are available upon request from the corresponding author. These data are not publicly available due to privacy restrictions according to the informed consent signed by the participants.

## Appendix A. Individual Factors Affecting Online Health Information-Seeking Behavior

**Paper**	**Individual Factors Affecting Online Health** **Information** **Information-** **Seeking Behavior**	**Methods of the Analysis**	**Limitations**
[14]	Gender Age Educational level Economic status (household income)	French nationally representative surveys and health barometers	Selection bias cannot be excluded. Results are not generalizable to other countries (different from France). Data on trust in the information found are only available for the sample of health information seekers.
[15]	Gender Age Educational level Economic status (household income)	Cross-sectional study	Fewer details on the online health information-seeking behavior of Chinese internet users. Results are difficult to compare with those of previous studies. The study has been conducted before the COVID-19 epidemic in 2020, during which the degree and diversity of internet use has grown.
[16]	Gender Age Educational level	Turkish Statistical Institute (TSI) survey	Exclusion of several additional independent variables, such as health status, the presence of chronic diseases, health literacy, and eHealth literacy.
[17]	Health status	Questionnaire survey	The external validity is reduced because the study is skewed toward younger and more educated patients. Self-reported health status and chronic medical condition is used in the study.
[11]	Health status	Queensland Social Survey	Results are based on self-reported data. Under-sampling of adults aged younger than 35 years. The topic of health information sought and the timing and frequency of online information seeking are not assessed. It is a cross-sectional study but the direction of association cannot be determined.
[10]	Gender Age Digital technology literacy	Sets of analyses on the 2012–2014 US Program for the International Assessment of Adult Competencies (PIAAC) Data using Stata	The study does not consider the effect of young adults’ digital literacy skills, problem-solving skills, and numeracy skills on their health-seeking approach. Lack of the ability to claim causation.
[12]	Age	Analysis of 10 e-commerce sites	The study only examines successful e-commerce sites in Turkey.
[13]	Age	Workshop-based discussions with the target group of 20 young drug users	Results are not generalizable to other countries (different from Slovenia).
[9]	Gender	Representative national German health survey	The cross-sectional data used does not allow for causal attributions. Differentiation is made between people who are searching for information for themselves and those who are searching for others.
[8]	Education	Focus groups	The transferability of findings is limited to populations similar to participants in the study.
[18]	Health status	Health Information National Trends Survey 4 Cycle	Results are not generalizable to other countries (different from the USA).
[19]	Education	Health Information National Trends Survey	The study examined three time periods but it does not allow for a comparison of rates across years. In all survey research, findings are limited by recall bias.

## Appendix B. Individual Factors Affecting Online Health Information Perception

**Paper**	**Individual Factors** **Affecting Online Health Information Perception**	**Methods of the Analysis**	**Limitations**
[21]	Race/ethnicity	Health Information National Trends Survey	The self-reported data are subject to both validity and bias issues. Collected data do not allow for longitudinal analysis of respondents. The sample does not help to capture nationally representative and generalizable trends. The model specification strategy has limited sample sizes.
[24]	Health literacy	Online survey	The number of samples is relatively small and only consists of people in China. The study failed to gain deep insights into how patient compliance changed with internet health information-seeking behavior. The used scales are more inclined to measure one’s trust in physicians.
[23]	Health literacy	Nationally representative middle-aged and older internet users	The theorized direction of relationships as portrayed can only be confirmed through the use of a longitudinal research design. Individuals who never used the Internet to locate health information were excluded from the analysis.
[22]	Health literacy	Online survey	The cross-sectional design of the study restricts the ability to infer causal relationships between health literacy and health information source preferences. The quality of the actual health information sources used by participants in the study is not been evaluated.
[25]	Health literacy, gender, and age	Online survey	The generalizability of results to various racial/ethnic minority groups is not possible.
[20]	Age, education, gender, and health status	A regional and population health survey	While the samples are of acceptable size, the cross-sectional design effectively prohibits a causal analysis of the relationships. The re-categorization of some of the dichotomous variables reduces the power of the estimates. Although this approach ensures a sufficient number of individuals in the final sample, it may also subject the survey to the element of self-selection, given that participation is voluntary.
